# Contemporary Approach to Postpartum Hemorrhage: Early Diagnosis and Evidence-Based Therapeutic Management

**DOI:** 10.7759/cureus.107095

**Published:** 2026-04-15

**Authors:** Eimy Zúñiga Gómez, Pablo R Durán Monge, Laura C Castro Rivero

**Affiliations:** 1 Gynecology, Caja Costarricense de Seguro Social (CCSS), San José, CRI; 2 Critical Care, Soporte Asistido Aéreo y Terrestre (SAAT), San José, CRI; 3 Gynecology, Hospital San Juan de Dios, San Jose, CRI

**Keywords:** hemorrhagic shock, massive transfusion, obstetric emergency, postpartum hemorrhage, uterine atony, uterotonics

## Abstract

Postpartum hemorrhage remains a major obstetric emergency and one of the most important contributors to maternal morbidity and mortality worldwide. Effective management depends on early recognition, systematic evaluation, and rapid implementation of evidence-based therapeutic strategies. A structured clinical approach facilitates identification of the underlying cause and supports timely intervention to prevent progression to severe hypovolemia and organ dysfunction. Risk assessment should begin during the antenatal period, as several maternal and obstetric conditions increase the likelihood of hemorrhagic complications. Intrapartum factors may further elevate risk, making continuous clinical vigilance essential throughout labor and delivery. Preventive measures, including appropriate management of the third stage of labor and prophylactic uterotonic therapy, represent key strategies for reducing the incidence and severity of hemorrhage. Early diagnosis relies on objective assessment of blood loss, continuous hemodynamic monitoring, and the use of structured clinical indicators that help identify deterioration at an early stage. Once hemorrhage is suspected, management requires immediate activation of institutional protocols, rapid hemodynamic stabilization, and a coordinated multidisciplinary response. Therapeutic interventions typically follow a stepwise escalation beginning with pharmacologic uterotonic agents and antifibrinolytic therapy, followed by mechanical measures and, when necessary, surgical procedures to control bleeding. Integration of standardized protocols, goal-directed transfusion strategies, and team-based management is essential to improve outcomes and reduce the burden of severe maternal complications associated with postpartum hemorrhage.

## Introduction and background

Postpartum hemorrhage remains the leading cause of maternal mortality worldwide and continues to impose a disproportionate burden on low- and middle-income countries (LMICs), where persistent limitations in healthcare infrastructure, delayed access to obstetric care, and constrained resource availability hinder timely and effective intervention [1,2]. Globally, an estimated 80,000 deaths are attributed to postpartum hemorrhage each year, with nearly 90% occurring in countries characterized by low socio-demographic indices [3]. This striking geographic inequality reflects not only disparities in access to skilled birth attendance and emergency obstetric services but also broader systemic shortcomings involving logistics, referral pathways, blood product availability, workforce capacity, and institutional preparedness. As a result, both the incidence and severity of postpartum hemorrhage are amplified in low-resource settings, where health systems frequently face difficulties in mounting rapid, coordinated, and evidence-based responses to obstetric emergencies [2].

Despite its substantial epidemiological impact, postpartum hemorrhage is also recognized as one of the most preventable causes of maternal death. A considerable proportion of fatal outcomes arises not from an intrinsic lack of therapeutic options but from delays in recognition, referral, escalation of care, and definitive management once bleeding has begun [2,3]. Inadequate implementation of standardized treatment pathways, inconsistent access to essential medications and transfusion support, and insufficient multidisciplinary coordination further contribute to poor outcomes, especially in vulnerable healthcare environments. Conversely, growing evidence indicates that the adoption of structured management protocols, early warning systems, and timely stepwise interventions can significantly reduce both maternal morbidity and mortality, particularly in LMICs where the margin for delay is especially narrow [2]. Therefore, efforts to reduce the burden of postpartum hemorrhage must extend beyond bedside management alone and also address the socioeconomic and structural inequities that continue to limit universal access to high-quality obstetric care [3,4].

At the same time, the clinical understanding of postpartum hemorrhage has evolved substantially in recent years, prompting important changes in both diagnostic definitions and therapeutic strategies. Traditional approaches relied predominantly on quantified blood loss thresholds, often without sufficiently accounting for the patient’s hemodynamic response or the dynamic progression of bleeding. More contemporary frameworks have moved toward a broader and clinically integrated interpretation that recognizes that fixed volumetric criteria may underestimate severity in selected patients and may delay intervention when compensatory mechanisms temporarily mask deterioration. This shift in perspective has encouraged greater emphasis on early recognition, rapid bedside assessment, and the integration of clinical signs with objective measurement strategies. In parallel, modern management paradigms prioritize the prompt use of uterotonic agents, early consideration of adjunctive therapies such as tranexamic acid, and the incorporation of point-of-care and laboratory-guided hemostatic evaluation to support individualized therapeutic decision-making. Balanced transfusion strategies and proactive correction of coagulopathy have also become increasingly central to contemporary care models aimed at preventing progression to hemorrhagic shock and multiorgan compromise [5].

Within this updated clinical paradigm, rapid recognition and activation of structured response systems are regarded as essential determinants of successful management. Delays in diagnosis, underestimation of blood loss, or failure to escalate care in a timely manner can precipitate severe maternal morbidity and substantially increase the risk of mortality [6,7]. For this reason, objective quantification of blood loss is now strongly advocated over visual estimation alone, given the well-documented inaccuracy of subjective assessment and its association with treatment delay [7]. Equally important is the implementation of standardized algorithms that facilitate coordinated escalation from initial resuscitative and pharmacologic measures to mechanical, surgical, and transfusion-based interventions when necessary. In this setting, multidisciplinary collaboration among obstetricians, anesthesiologists, hematologists, nursing personnel, and blood bank services plays a critical role in ensuring consistency of care and optimizing maternal outcomes [5].

The objective of this review is to comprehensively examine the contemporary approach to postpartum hemorrhage by integrating current epidemiological evidence, evolving diagnostic definitions, and structured management strategies, with a particular emphasis on early recognition and evidence-based therapeutic interventions.

Methodology

This was developed as a structured narrative review aimed at providing an updated and clinically integrated analysis of postpartum hemorrhage, with particular emphasis on early diagnosis, risk stratification, and evidence-based therapeutic management. The review was conducted in accordance with the SANRA (Scale for the Assessment of Narrative Review Articles) framework and followed a predefined methodological approach established prior to literature screening. Given the clinical heterogeneity of postpartum hemorrhage across obstetric settings, the variability in definitions, and the time-sensitive nature of therapeutic interventions, a narrative interpretative synthesis was selected over quantitative pooling in order to integrate epidemiologic trends, evolving diagnostic criteria, and stepwise management strategies into a coherent and clinically applicable framework [8].

A comprehensive literature search was conducted in PubMed, Scopus, and Web of Science, including peer-reviewed articles published in English or Spanish between January 2020 and December 2025. The final search was performed in March 2026. This timeframe was selected to capture contemporary advances in obstetric hemorrhage bundles, early warning systems, quantitative blood loss measurement, tranexamic acid use, massive transfusion protocols, viscoelastic-guided hemostatic management, and updated international recommendations for postpartum hemorrhage care. Foundational studies were incorporated when necessary to contextualize pathophysiological mechanisms, landmark clinical trials, and the evolution of contemporary postpartum hemorrhage definitions.

The search strategy combined Medical Subject Headings (MeSH) terms and free-text keywords with Boolean operators related to postpartum hemorrhage, obstetric hemorrhage, uterine atony, retained placenta, genital tract trauma, coagulopathy, shock index, quantitative blood loss, uterotonics, tranexamic acid, balloon tamponade, compression sutures, uterine artery embolization, massive transfusion, fibrinogen replacement, viscoelastic testing, and maternal outcomes. Because this article was conceived as a structured narrative review rather than a systematic review, exact database-specific search strings are not presented; however, the principal search domains and representative terms were defined prospectively to support methodological transparency. 

Inclusion criteria comprised randomized controlled trials, large observational cohorts, systematic reviews, meta-analyses, expert consensus statements, and contemporary international guidance addressing early recognition, risk stratification, diagnostic evaluation, therapeutic interventions, transfusion strategies, and maternal outcomes in postpartum hemorrhage. Exclusion criteria included non-peer-reviewed publications, isolated case reports, editorials without outcome data, purely technical procedural descriptions lacking maternal outcome measures, redundant datasets, and studies not directly addressing clinically relevant diagnostic or therapeutic aspects of postpartum hemorrhage.

For study selection purposes, postpartum hemorrhage was operationally defined as cumulative blood loss of at least 1000 mL or any amount of bleeding associated with signs of hypovolemia within the first 24 hours postpartum, in accordance with contemporary clinically integrated definitions [1]. Studies using alternative volumetric thresholds were also considered when clinical severity criteria were clearly described, and definitional variability was taken into account during the interpretative synthesis.

Priority was assigned to multicenter investigations, studies applying standardized postpartum hemorrhage definitions, and research evaluating predefined primary outcomes, including maternal mortality, severe maternal morbidity, need for hysterectomy, transfusion requirements, and escalation to invasive procedures. Secondary outcomes included intensive care unit admission, duration of hospitalization, and treatment-related complications. Extracted variables comprised study design, setting, and resource level, patient risk profile, etiology of hemorrhage, diagnostic approach, including quantitative blood loss methods and hemodynamic indices, timing and type of intervention, transfusion and hemostatic strategies, and reported clinical endpoints. Reference lists of included studies were manually screened to identify additional relevant publications.

The initial search yielded 212 records. After removal of duplicates, 168 articles remained for title and abstract screening. Of these, 97 underwent full-text evaluation, and 54 studies were included in the final synthesis. Selection was performed independently by two authors, with disagreements resolved through discussion and consensus. Because this was developed as a narrative review, formal inter-reviewer agreement metrics such as Cohen’s kappa were not calculated. 

Methodological quality and internal validity were assessed narratively. Randomized trials were appraised with attention to allocation methods and general risk of bias domains, observational studies were evaluated considering cohort selection, confounding adjustment, and follow-up completeness, and systematic reviews were assessed according to methodological transparency and consistency of inclusion criteria. Greater interpretative weight was assigned to higher-level evidence, multicenter investigations, and guideline-supported recommendations in cases of conflicting findings. Contextual heterogeneity, including differences between high-resource and low-resource settings, was explicitly considered during synthesis to preserve clinical applicability across diverse healthcare systems. Given its narrative design, this review is subject to potential selection bias, language bias due to restriction to English- and Spanish-language studies, and the inherent subjectivity of interpretative synthesis, and it does not provide pooled quantitative effect estimates.

## Review

Definitions, classification, and pathophysiological mechanisms

Postpartum hemorrhage is currently defined in quantitative terms as a cumulative blood loss of at least 1000 mL or any degree of bleeding associated with clinical signs of hypovolemia, reflecting a shift toward objective measurement rather than reliance on visual estimation, which has consistently been shown to be inaccurate [7]. This updated definition underscores the importance of systematic assessment and timely recognition, as underestimation of blood loss may delay critical interventions. In addition to volumetric criteria, postpartum hemorrhage is classified according to its timing, distinguishing primary from secondary forms. Primary postpartum hemorrhage occurs within the first 24 hours following delivery, whereas secondary postpartum hemorrhage develops between 24 hours and 12 weeks postpartum, a distinction that facilitates etiologic evaluation and guides diagnostic and therapeutic decision-making [9].

Understanding this classification requires consideration of the physiological mechanisms that normally ensure hemostasis after childbirth. Following placental separation, effective uterine contraction compresses the spiral arteries and promotes vascular occlusion, thereby limiting blood loss. When this physiological process is disrupted, bleeding may become excessive and progress to postpartum hemorrhage [9]. Within this context, the etiologic framework commonly described as the “4 Ts” (tone, tissue, trauma, and thrombin) provides a structured approach to identifying underlying causes. Tone, referring to uterine atony, represents the leading cause of postpartum hemorrhage and accounts for approximately 70-80% of cases, occurring when the uterus fails to contract adequately after delivery [10]. Tissue denotes retained placental fragments, which can mechanically interfere with uterine contraction and perpetuate bleeding; this mechanism is particularly relevant in cases of placenta accreta spectrum, in which abnormal placental invasion into the uterine wall complicates separation and removal [11]. Trauma encompasses genital tract lacerations, uterine rupture, and hematomas, all of which may contribute significantly to hemorrhage and often require prompt surgical intervention [10]. Thrombin refers to inherited or acquired coagulopathies that impair effective clot formation and exacerbate bleeding, highlighting the necessity of early identification and targeted correction of coagulation abnormalities [12].

When hemorrhage is severe or rapidly progressive, the pathophysiological cascade may culminate in obstetric hemorrhagic shock. Acute and substantial blood loss reduces circulating volume, leading to hypotension, diminished tissue perfusion, and the risk of multiorgan dysfunction if not promptly addressed [10]. In this setting, immediate resuscitative measures and definitive hemostatic interventions are essential to prevent further deterioration. Moreover, the management of severe postpartum hemorrhage may itself influence coagulation dynamics. Massive transfusion protocols, while lifesaving, can contribute to dilutional coagulopathy through the replacement of red blood cells without proportional administration of clotting factors, whereas ongoing uncontrolled bleeding may precipitate consumptive coagulopathy due to progressive depletion of coagulation components [13].

Risk assessment and preventive strategies

Antenatal and intrapartum factors play a central role in the stratification of risk for postpartum hemorrhage, as several maternal and obstetric conditions have been consistently associated with an increased likelihood of excessive bleeding. Among antenatal risk factors, multiple gestation, polyhydramnios, maternal anemia, and a prior history of postpartum hemorrhage are particularly relevant, as each of these conditions may predispose to uterine overdistension, impaired myometrial contractility, or reduced physiological reserve in the event of blood loss [14]. These preexisting factors not only increase susceptibility to hemorrhage during and after delivery but also underscore the importance of anticipatory planning and enhanced surveillance in high-risk pregnancies. In addition to antenatal conditions, several intrapartum factors significantly contribute to the development of postpartum hemorrhage. Prolonged labor, intra-amniotic infection such as chorioamnionitis, and operative delivery have been identified as key contributors, largely due to their association with uterine atony, the primary cause of postpartum hemorrhage. Prolonged or dysfunctional labor may exhaust uterine musculature, while infection-related inflammation can impair effective myometrial contraction. Similarly, operative interventions may increase the risk of trauma or compromise normal uterine physiology, thereby amplifying hemorrhagic risk. Recognizing these intrapartum elements is essential for early intervention and escalation of preventive measures [14].

Placenta previa and placenta accreta spectrum disorders represent particularly high-risk conditions within this framework. Both are characterized by abnormal placental implantation and separation dynamics, which can lead to severe bleeding during delivery due to impaired placental detachment and exposure of large uteroplacental vessels [14]. 

In this context, antenatal imaging assumes a critical role in risk identification. The timely detection of placenta previa and placenta accreta spectrum disorders through imaging modalities enables appropriate delivery planning and the anticipation of potential complications. Based on identified risks, decisions regarding the timing, location, and mode of delivery can be strategically adjusted to minimize hemorrhagic complications and ensure the availability of surgical expertise and blood products when needed [14].

Preventive strategies extend into the intrapartum period, particularly through the active management of the third stage of labor. The administration of uterotonic agents, most commonly oxytocin, immediately after neonatal delivery and prior to placental expulsion is recommended as a core preventive measure. This intervention promotes effective uterine contraction and reduces the likelihood of excessive postpartum bleeding. In addition to oxytocin, prophylactic uterotonics such as carbetocin and combinations including tranexamic acid or misoprostol have demonstrated efficacy in reducing the risk of postpartum hemorrhage [15,16]. The timing and dosing of these agents are critical, with administration recommended immediately after delivery to optimize uterine tone. Although not universally available, carbetocin has been recognized for its effectiveness as a single-agent therapy, particularly in settings where sustained uterotonic activity is desirable [16].

Beyond individual pharmacologic measures, institutional preparedness is fundamental to effective risk mitigation. The implementation of standardized protocols and structured risk stratification models allows healthcare teams to identify and proactively manage patients at elevated risk of postpartum hemorrhage. Tools such as three-tiered risk assessment systems categorize patients into low-, medium-, and high-risk groups, thereby facilitating tailored monitoring and intervention strategies [17]. Complementary approaches, including the calculation of Maximum Allowable Blood Loss and the use of point-of-care coagulation monitoring, further enhance individualized care by enabling early recognition of decompensation and guiding timely therapeutic adjustments [18].

Early diagnosis and clinical monitoring

The estimation of blood loss during and after delivery has traditionally relied on visual assessment; however, visual estimation of blood loss remains highly inaccurate and frequently underestimates actual hemorrhage volume by as much as 40% [7,19]. This systematic underestimation contributes directly to delayed recognition of postpartum hemorrhage and postponement of therapeutic escalation, thereby increasing the risk of severe maternal outcomes [20]. The limitations of visual assessment underscore the need for more objective and reproducible measurement strategies capable of triggering timely intervention. In response to these shortcomings, quantitative blood loss measurement techniques have been increasingly advocated. The gravimetric method, which involves weighing blood-soaked materials and subtracting their dry weight, provides a more accurate estimate than visual approximation, although it may be labor-intensive and require logistical coordination [20]. Alternatively, the volumetric method employs calibrated drapes or collection bags to directly measure blood loss, offering greater reliability and reducing the likelihood of underestimation [21]. Calibrated drapes, in particular, allow for real-time collection and objective quantification of bleeding during delivery, facilitating earlier recognition of excessive blood loss and more consistent clinical decision-making [7]. 

Despite improved measurement techniques, clinical evaluation remains essential. Early signs of hypovolemia typically include tachycardia and hypotension; however, these manifestations may not become apparent until substantial blood loss has already occurred. Moreover, young and otherwise healthy patients often exhibit significant compensatory capacity, temporarily maintaining blood pressure and perfusion despite ongoing hemorrhage. This physiological compensation can mask early deterioration and delay recognition until a critical threshold is reached. Consequently, reliance solely on traditional vital signs may be insufficient for timely diagnosis [19].

To enhance predictive accuracy, the obstetric shock index, which incorporates the relationship between heart rate and systolic blood pressure, has been proposed as a useful tool for assessing hemorrhage severity and guiding escalation of care [9]. In parallel, predictive models and risk scores integrating clinical variables such as pre-eclampsia and duration of labor have been developed to identify women at increased risk of postpartum hemorrhage [22]. 

Laboratory evaluation further refines assessment by providing objective indicators of physiological compromise. Serial monitoring of hemoglobin levels and lactate concentrations can offer insight into the magnitude of blood loss and the adequacy of compensatory mechanisms. Among coagulation parameters, fibrinogen has emerged as an early marker of coagulopathy in postpartum hemorrhage, with low levels correlating with severe bleeding and increased transfusion requirements [12]. 

The integration of clinical and laboratory data into structured early warning scores has further strengthened diagnostic vigilance in obstetric practice. These scoring systems synthesize hemodynamic parameters and biochemical markers to facilitate early detection of clinical deterioration and prompt escalation of care [7]. By standardizing thresholds for intervention, early warning scores contribute to more consistent and timely management, thereby reducing the likelihood of severe maternal outcomes [23].

Ultimately, the effective management of postpartum hemorrhage requires continuous reassessment and dynamic monitoring. Ongoing evaluation of clinical status, hemodynamic parameters, and laboratory findings is essential to detect subtle changes in patient condition and to adjust treatment strategies accordingly [9]. Dynamic monitoring enables the timely implementation of interventions such as blood transfusion and hemostatic support, preventing progression to advanced hemorrhage and associated complications [5].

Initial stabilization and hemodynamic resuscitation

The immediate management of postpartum hemorrhage requires a structured and systematic approach grounded in clearly defined early priorities. Central to this framework are the “Four Early Principles,” which emphasize early call for help, early comprehensive assessment, early hemostasis directed at the underlying cause, and early volume resuscitation with component transfusion in order to prevent severe complications and maternal mortality [9]. These principles reflect the time-sensitive nature of obstetric hemorrhage and underscore the necessity of prompt, coordinated action. In this context, activation of obstetric emergency response systems or hemorrhage protocols is essential to ensure rapid mobilization of personnel, availability of resources, and effective communication among healthcare providers [24]. 

A fundamental component of this initial response is the prompt establishment of large-bore intravenous access to enable rapid administration of fluids and blood products. Adequate vascular access is critical for effective resuscitation in the setting of hypovolemic shock secondary to postpartum hemorrhage [9]. Fluid resuscitation strategies typically begin with crystalloids; however, early replacement with blood products is strongly emphasized, particularly when ongoing bleeding or hemodynamic instability is present. Massive transfusion protocols have therefore become integral to contemporary management, supporting balanced component therapy. In this setting, point-of-care assessments such as thromboelastography facilitate rapid evaluation of coagulation status and help guide targeted transfusion strategies [10].

Within hemodynamic management, careful consideration must be given to the balance between permissive hypotension and aggressive resuscitation. Although aggressive volume replacement may restore blood pressure and perfusion, it must be judiciously administered to avoid worsening dilutional coagulopathy and exacerbating bleeding complications. Therefore, resuscitation strategies must be tailored to the individual clinical scenario, maintaining perfusion while minimizing secondary physiological harm [6].

Parallel to hemodynamic stabilization, preventing the development of the so-called lethal triad-hypothermia, acidosis, and coagulopathy-is a critical objective in postpartum hemorrhage management. Hypothermia impairs coagulation function, acidosis reduces the efficacy of clotting factors, and coagulopathy further perpetuates bleeding. Early recognition and correction of coagulation abnormalities are therefore essential, including the timely administration of tranexamic acid and fibrinogen concentrate when indicated. Addressing these interrelated physiological derangements reduces the likelihood of progression to massive hemorrhage and multiorgan compromise [5].

Effective implementation of these interventions depends on coordinated multidisciplinary collaboration. The management of postpartum hemorrhage frequently requires the involvement of obstetricians, anesthesiologists, hematologists, and, in selected cases, acute care surgeons. Structured multidisciplinary training programs and clearly defined roles have been associated with improved maternal outcomes and reduced mortality [25]. The OBS Cymru project provides a practical illustration of this approach, demonstrating that a comprehensive care bundle incorporating risk assessment, quantitative blood loss measurement, and structured escalation to senior clinicians resulted in a reduction in the incidence of massive postpartum hemorrhage and red blood cell transfusion [23].

Pharmacologic management based on evidence

Pharmacologic management constitutes a central pillar in the prevention and treatment of postpartum hemorrhage, with oxytocin remaining the first-line uterotonic agent in the United States. It is typically administered intravenously at a dose of 10 IU immediately after delivery of the neonate, as part of active management of the third stage of labor [15]. This early administration promotes effective uterine contraction and reduces the likelihood of excessive bleeding. Emerging evidence suggests that dosing strategies may influence clinical outcomes, as high-dose oxytocin regimens of 80 units have been associated with a significant reduction in postpartum hemorrhage rates when compared with lower-dose regimens ranging from 10 to 30 units, indicating that higher doses may confer greater preventive efficacy in selected populations [26]. 

When oxytocin alone proves insufficient to achieve adequate uterine tone, second-line uterotonic agents are introduced in a stepwise manner. Methylergometrine, carboprost, and misoprostol are commonly employed in this context, either sequentially or in combination with oxytocin to produce additive or synergistic effects [16]. Combination therapy has been shown to reduce the risk of postpartum hemorrhage more effectively than oxytocin monotherapy. In particular, the addition of misoprostol to oxytocin has demonstrated a significant reduction in postpartum hemorrhage rates and composite maternal morbidity among high-risk patients [27]. 

The selection of second-line agents requires careful consideration of comparative effectiveness and patient-specific contraindications. Methylergometrine is effective in promoting sustained uterine contraction but may induce hypertension, rendering it contraindicated in patients with pre-existing hypertensive disorders. Carboprost is also effective; however, it is associated with gastrointestinal side effects and should be used cautiously in patients with asthma due to its bronchoconstrictive potential [16]. Misoprostol represents a practical and accessible alternative, particularly in low-resource settings, given its ease of administration, stability at room temperature, and cost-effectiveness [28]. 

Beyond uterotonic therapy, antifibrinolytic treatment has assumed an increasingly prominent role. Tranexamic acid has demonstrated efficacy in reducing maternal mortality and decreasing the need for blood transfusion in postpartum hemorrhage, and ongoing investigation through the WOMAN-2 (World Maternal Antifibrinolytic Trial-2) trial is evaluating its prophylactic use in women with moderate to severe anemia [16]. Early administration of tranexamic acid is recommended in cases of ongoing or refractory hemorrhage to enhance hemostasis and limit further blood loss [10]. 

In cases of persistent bleeding associated with coagulopathy, fibrinogen concentrate and other adjunctive hemostatic agents may be required to restore effective clot formation. These therapies are typically integrated into comprehensive management plans that include blood product replacement and activation of massive transfusion protocols when indicated [10]. Such measures address both the mechanical and hematologic components of hemorrhage.

Overall, an algorithm-based approach to pharmacologic escalation is recommended to ensure structured and timely intervention. This strategy begins with oxytocin and progresses to second-line uterotonics and adjunctive therapies as clinically indicated. By adhering to predefined escalation pathways, clinicians can reduce treatment delays, standardize care, and minimize the risk of severe complications associated with uncontrolled postpartum hemorrhage [9].

Mechanical and minimally invasive interventions

Mechanical interventions play a central role in the stepwise management of postpartum hemorrhage, particularly in cases of uterine atony, which remains the most common cause of severe bleeding. Uterine massage and bimanual compression are considered first-line mechanical maneuvers in this setting because they aim to stimulate myometrial contraction and mechanically reduce uterine blood loss. These interventions are typically performed concurrently with uterotonic therapy in order to enhance uterine tone and promote hemostasis. In some cases, this combined approach is sufficient to control bleeding; however, persistent hemorrhage despite these initial measures requires escalation to more advanced interventions [29].

When bleeding remains refractory to pharmacologic treatment and manual compression, intrauterine balloon tamponade is widely regarded as an appropriate next step. This intervention achieves hemostasis by exerting direct pressure against the uterine walls and is most commonly used when hemorrhage does not respond adequately to uterotonics and uterine massage. Reported success rates generally range from approximately 80% to 90%, although effectiveness varies according to the underlying etiology, timing of placement, and clinical setting. Higher success rates have been described in cases of uterine atony than in hemorrhage related to placental implantation abnormalities [30]. In addition, early insertion of intrauterine balloon tamponade, ideally soon after identification of refractory bleeding, has been associated with lower blood loss and improved clinical outcomes [31].

In parallel with balloon-based approaches, vacuum-induced uterine tamponade systems have emerged as promising alternatives. Devices such as the Jada System function by applying negative pressure within the uterine cavity, thereby facilitating uterine collapse and promoting hemostasis in a manner that more closely resembles physiologic uterine contraction [32]. Preliminary evidence suggests that these vacuum-induced systems may reduce blood loss and transfusion requirements in selected patients when compared with traditional balloon tamponade; however, further studies are still needed to better define their comparative effectiveness and their role within standardized management algorithms [33].

If hemorrhage persists despite mechanical tamponade, radiologic intervention may be considered in appropriately selected patients. Uterine artery embolization is a minimally invasive uterine-sparing procedure designed to reduce arterial perfusion to the bleeding source. It is generally reserved for cases in which less invasive measures have failed and is particularly useful in patients who are sufficiently stable to undergo interventional radiologic management. Reported success rates are generally high, most commonly ranging from approximately 85% to 95%, although outcomes vary according to hemorrhage severity, etiology, timing of intervention, and local expertise [29].

Escalation to surgical management becomes necessary when mechanical and minimally invasive strategies fail to achieve hemostasis or when the patient remains hemodynamically unstable. Surgical options may include uterine compression sutures, stepwise devascularization procedures, or, in more severe and refractory cases, hysterectomy. The choice of procedure depends on the clinical scenario, the etiology of hemorrhage, the patient’s hemodynamic status, and operator experience. Whenever feasible, uterine-sparing techniques are preferred in order to preserve future fertility; however, definitive surgical intervention is warranted when maternal stabilization cannot otherwise be achieved [34].

Surgical management and advanced interventions

When conservative medical and mechanical measures fail to control postpartum hemorrhage, uterine-sparing surgical techniques are often employed as the next step in escalation. Among these, uterine compression sutures represent one of the most widely adopted and effective interventions. The B-Lynch suture is among the best-known techniques and consists of the placement of sutures around the uterus in order to provide continuous compression and achieve hemostasis. Over time, several technical modifications have been introduced with the aim of improving efficacy and reducing operative complexity. Modified approaches, including knapsack-like sutures and other variations of the B-Lynch technique, have shown favorable outcomes in selected series and may offer procedural advantages such as shorter operative time and fewer postoperative complications when compared with the classic method [35,36].

More broadly, published data indicate that uterine compression sutures are associated with generally high success rates across studies, most commonly ranging from approximately 75% to 95%, with several reports and reviews describing effectiveness near or above 90%, depending on hemorrhage etiology, timing of intervention, and operator experience [34]. Despite their favorable success profile, these procedures are not without risk. Intrauterine synechiae and other postoperative uterine sequelae have been described, with adhesions reported in a minority of cases, underscoring the importance of appropriate postoperative surveillance and long-term follow-up when fertility preservation is intended [34].

If bleeding persists despite compression sutures, stepwise uterine devascularization may be undertaken. This strategy involves sequential ligation of the uterine vasculature to reduce uterine perfusion and control hemorrhage. Techniques such as O'Leary’s bilateral uterine artery ligation and Tsirulnikov’s triple ligation are commonly used within this framework. These procedures have demonstrated high success rates, often reported around 90%, and are generally associated with low complication rates, making them an important component of fertility-preserving surgical management [34].

In more refractory cases, internal iliac artery ligation may be considered. This procedure aims to reduce pelvic arterial pressure and blood flow, but it is technically more demanding than uterine artery ligation and generally less favored when other uterine-sparing options are feasible. Reported success rates are variable and are often lower than those of uterine artery embolization, while the procedure also carries the possibility of severe complications because of its anatomical complexity. For these reasons, internal iliac artery ligation is usually reserved for selected cases in which other devascularization techniques have failed, are unavailable, or are not feasible in the clinical context [34].

When all conservative and uterine-sparing measures are unsuccessful, and the patient remains hemodynamically unstable, emergency obstetric hysterectomy becomes the definitive intervention. This procedure is considered a last-resort, life-saving measure, particularly in cases of severe uterine atony, uncontrolled hemorrhage, or placenta accreta spectrum disorders [37,38]. Hysterectomy is generally performed when ongoing bleeding cannot be controlled through less invasive strategies and maternal instability persists [34]. Although it is highly effective in achieving definitive hemostasis, it is associated with substantial maternal and neonatal morbidity, which largely reflects the severity and urgency of the underlying clinical scenario [38].

In contrast, uterine-sparing procedures such as compression sutures and uterine artery ligation are generally not associated with major impairment of future fertility, although long-term reproductive outcomes remain incompletely defined and warrant further study [34]. Following any major surgical intervention for postpartum hemorrhage, vigilant postoperative critical care is essential. Postoperative management should include close monitoring for complications such as infection, recurrent bleeding, and thromboembolic events, along with continued hemodynamic reassessment and blood product support when necessary [10]. Early recognition of postoperative deterioration and dynamic monitoring remain fundamental to preventing secondary complications and improving overall maternal outcomes [9].

Transfusion strategies, systems-based care, and future directions

In cases of severe postpartum hemorrhage, massive transfusion protocols constitute a cornerstone of advanced resuscitative management. These protocols emphasize the early and balanced administration of red blood cells, plasma, and platelets in order to prevent or correct coagulopathy while stabilizing hemodynamic status [6, 10]. The rationale underlying this strategy is the recognition that hemorrhagic shock is not solely a problem of volume depletion but also of progressive coagulation impairment. Balanced component therapy, characterized by the use of equal or near-equal ratios of blood components, has been shown to improve outcomes by addressing the dynamic and specific coagulation needs that arise during ongoing hemorrhage [6]. 

Within this framework, fibrinogen plays a particularly critical role. As one of the first coagulation factors to decline during postpartum hemorrhage, low fibrinogen levels are closely associated with severe bleeding and adverse outcomes. Maintaining fibrinogen concentrations above 2 g/L is considered essential to prevent progression of hemorrhage and worsening coagulopathy [13,39]. Rather than relying exclusively on empirical transfusion strategies, fibrinogen replacement guided by viscoelastic testing allows for more precise correction of deficiencies and may reduce unnecessary exposure to blood products [39,40]. 

The increasing adoption of viscoelastic hemostatic assays, including thromboelastography and rotational thromboelastometry, has further advanced goal-directed transfusion therapy. These point-of-care tests provide rapid assessment of the entire coagulation process, from clot initiation to clot stability, enabling clinicians to identify specific coagulation defects in real time [41]. By guiding the selective administration of plasma, platelets, or fibrinogen concentrate based on measured deficiencies, viscoelastic testing supports more efficient transfusion practices and has been associated with reduced morbidity and lower overall blood product utilization [36,39]. 

Beyond individual therapeutic interventions, system-level strategies have demonstrated a significant impact on maternal outcomes. Standardized care pathways, including obstetric hemorrhage bundles, promote consistency and coordination in postpartum hemorrhage management by ensuring early recognition, rapid response, and structured escalation of care. These bundles commonly incorporate predefined protocols for communication, transfusion activation, and multidisciplinary collaboration, thereby reducing variability in practice and minimizing treatment delays [5,6]. 

Effective implementation of these structured approaches depends on preparedness and team competency. Simulation training and team-based drills are therefore essential components of contemporary postpartum hemorrhage management. Through repeated practice of standardized protocols in simulated emergency settings, healthcare providers improve communication, role clarity, and coordination under pressure. These exercises enhance overall readiness and facilitate the seamless execution of hemorrhage response algorithms when real events occur [6].

In low-resource settings, adaptation of these strategies requires pragmatic modification to align with available infrastructure. Efficient use of existing resources, simplification of protocols, and prioritization of essential interventions allow for implementation even in environments with limited equipment or blood product availability. In such contexts, focused training and education are particularly critical to ensure that healthcare providers can recognize postpartum hemorrhage promptly and initiate appropriate management despite systemic constraints [6].

Looking forward, emerging technologies offer additional opportunities to refine prevention and response. Predictive analytics and data-driven risk assessment tools hold promise for the early identification of patients at elevated risk for postpartum hemorrhage, potentially enabling preemptive interventions before clinical deterioration occurs. By integrating patient-specific data into decision-making processes, these technologies may enhance anticipatory care and further reduce morbidity associated with obstetric hemorrhage [6,41].

## Conclusions

Postpartum hemorrhage represents a dynamic and multifactorial obstetric emergency in which updated quantitative definitions, structured classification, and a clear understanding of pathophysiological mechanisms are fundamental for timely recognition and intervention. The integration of objective blood loss measurement, risk stratification models, early warning systems, and laboratory-guided monitoring strengthens diagnostic accuracy and reduces delays in care. When combined with protocol-driven initial stabilization, balanced transfusion strategies, and targeted correction of coagulopathy, this comprehensive approach addresses both the mechanical and hemostatic components of hemorrhage. Early activation of multidisciplinary response systems and adherence to standardized escalation pathways are critical determinants of improved maternal outcomes.

Effective management of postpartum hemorrhage requires a stepwise, evidence-based strategy that progresses from preventive uterotonic administration and mechanical interventions to advanced surgical and transfusion therapies when necessary. Uterine-sparing techniques, minimally invasive procedures, and goal-directed transfusion guided by viscoelastic testing allow for individualized care while preserving fertility whenever possible. System-based measures, including hemorrhage bundles, simulation training, and adaptation to resource-limited settings, further enhance preparedness and response efficiency. Collectively, the integration of clinical vigilance, structured protocols, and multidisciplinary coordination remains essential to reducing severe maternal morbidity and mortality associated with postpartum hemorrhage.
